# A landscape analysis of perinatal mental health delivery among stakeholders in Ecuador and Peru

**DOI:** 10.1017/gmh.2025.10090

**Published:** 2026-02-19

**Authors:** Oriana Culbert, Itziar Familiar-Lopez, Joaquín Castro Vergara, Nergiz Turgut, María Sol Garcés Espinosa, Victor Orlando Cruz, Elizabeth Foot, Natalia Halpern Lagos, Angela Marie Johnson, Maria Muzik, Gwenyth O. Lee

**Affiliations:** 1 Rutgers Global Health Institute, Rutgers The State University of New Jersey, USA; 2 Department of Psychiatry, Michigan State University, USA; 3 Instituto de Neurociencias, Universidad San Francisco de Quito, Ecuador; 4 Colegio de Ciencias Sociales y Humanidades, Carrera de Psicología, Universidad San Francisco de Quito, Ecuador; 5 Universidad de San Martín de Porres, Peru; 6 University of Michigan, USA; 7 Department of Psychiatry, University of Michigan-Michigan Medicine, USA; 8 Department of Psychiatry, University of Michigan-Michigan Medicine and Program for Multicultural Health, Department of Community Health Services, University of Michigan Health, Michigan Medicine, USA

**Keywords:** perinatal, mental health, barriers, health services, global mental health

## Abstract

Perinatal mental health disorders are prevalent in Ecuador and Peru. Despite national health policies supporting maternal mental health care, service provision remains fragmented, relying on a mix of public, private, and nongovernmental actors. This study examined professional interest holders’ perceptions of barriers to perinatal mental health care and the solutions they propose. We employed a mixed-methods approach. First, a systematic review of publicly available data was conducted to identify organizations engaged in maternal and mental health care in Ecuador and Peru. Following this, in-depth, semistructured interviews were conducted with 17 key informants representing research institutions, nongovernmental organizations (NGOs), government agencies, and private sector entities. Thematic analysis was applied to identify structural barriers, institutional challenges, and proposed solutions. Findings revealed multilevel barriers to perinatal mental health care, including stigma, financial constraints, limited provider training, fragmented health services, and bureaucratic inefficiencies. Community-based interventions, task-shifting strategies, and increased public education were identified as effective approaches to addressing these challenges. Participants also emphasized the need for intersectoral collaboration, increased governmental investment, and policy reforms to strengthen maternal mental health services. Efforts to improve perinatal mental health care in Ecuador and Peru require a combination of culturally sensitive, community-driven interventions, as well as sustainable government investment and commitment.

## Graphical abstract

**Figure:**
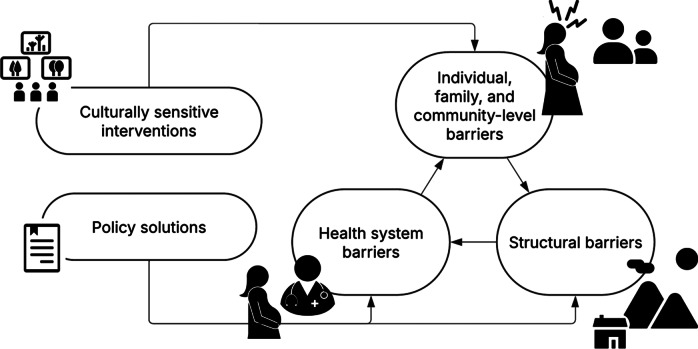

## Impact statement

This study sheds light on the barriers that providers face when promoting perinatal mental health services in Ecuador and Peru. The study evaluates how systemic factors, alongside stigma and cultural expectations make it difficult to deliver care, and how these same factors shape the experiences of women seeking support. Despite these challenges, providers identified community-driven strategies like task-shifting and culturally responsive care as promising ways to expand access. Their insights can help shape stronger mental health systems and guide efforts to support perinatal mental health, both in the region and beyond.

## Introduction

Perinatal mental health disorders include a range of emotional and mental health issues that can occur (Stevenson et al., 2023). These disorders adversely affect the well-being of the mother and are also associated with various adverse outcomes for the child. In high-income countries (HICs), this includes behavioral and emotional challenges (Muzik and Borovska, 2011). In low- and middle-income countries (LMICs), poor perinatal mental health has been associated with an increased risk of child mortality, as well as an increased risk of undernutrition and poorer child development (Surkan et al., 2014; Leis et al., 2014; Boivin et al., 2018).

Depression is one of the most common perinatal mental health disorders (Roddy Mitchell et al., 2023). In LMICs, around 15.6% of pregnant women and 19.8% of postpartum women experience perinatal depression (World Health Organization, n.d.). In Latin America, these may be even higher, as a recent systematic review found a pooled prevalence of 25.3% for antenatal depression and 19.0% for postpartum depression (Rondon, 2020). Disparities in the prevalence of depression between HICs and LMICs are shaped by socioeconomic factors, healthcare access, and cultural differences (Rondon, 2020). In addition, recent challenges across Latin America have exacerbated perinatal mental health challenges, with studies reporting increased rate of depression and anxiety among pregnant and postpartum women following the COVID-19 pandemic (Iyengar et al., 2021), which likely also exacerbated other significant structural risk factors for poorer mental health, including poverty (Rondon, 2020; Ghahyazi et al., 2023; Miño and Flores, 2023), displacement (Anderson et al., 2017; Stevenson et al., 2023), intimate partner violence (Rondon, 2020; Meneses et al., 2024) and exposure to natural disasters. These stressors, alongside other factors such as limited mental health resources (Rondon, 2020) and societal stigma (Torri, 2013), also create significant barriers to accessing necessary support services. However, it has also been reported that the COVID-19 pandemic has led to increasing discussion of mental health issues by researchers, implementers, and the public, resulting in the rapid development and deployment of new strategies by public and private actors to support mental health in the region better.

Ecuador and Peru are countries in Andean South America where, although maternal mental health services are theoretically supported through the public healthcare system, delivery often relies on a fragmented network of public and private providers, NGOs, and employers, leading to inconsistent access to care. A study analyzing data from the 2018 National Health and Nutrition Survey (ENSANUT) in Ecuador found that 15% of women surveyed experienced postpartum depression (Parra-parra et al., 2022), while in Peru, studies show that between 24% and 40% of pregnant women experience perinatal depression (Aramburú et al., 2008; Luna Matos et al., 2009).

In both countries, mental health care has been periodically promoted within the national health agenda with inconsistent success. In Ecuador, although the Ministry of Public Health created policies to integrate mental health into primary healthcare in the early 2000s, implementation was hindered by limited resources and a shortage of qualified professionals (Maloney, 2022). As a result, over the 2010s to early 2020s, indicators of access to care for people with mental health stagnated (from 5.3 mental health workers per 100,000 individuals in 2014 to 7.2 per 100,000 in 2020) (World Health Organization, 2020). Similarly, in the early 2010s, Peru pursued efforts to decentralize mental health services, and expand the delivery of mental health support interventions from tertiary-level systems to the primary care level (Miranda et al., 2017; Keynejad et al., 2021), followed by legal reforms to guarantee the rights of people with mental health conditions (Toyama et al., 2017). Coinciding with a period of economic and social stability, these actions have led to increases in access to care for people with mental health problems (from 11% in 2012 to 26% in 2018) (Castillo-Martell and Cutipé-Cárdenas, 2019), and 32 mental health workers per 100,000 individuals available in 2020 – although still falling short of national targets (World Health Organization, 2017; Villarreal-Zegarra et al., 2023).

There have also been periodic efforts to integrate mental health support into maternal services. For example, in Ecuador, ‘Establecimientos Amigos de la Madre y del Niño’ (ESAMyN) certification, modeled after the global Baby-Friendly Hospital initiative, promotes health education and social support for women during their pregnancies. Although ESAMyN has proved beneficial, its expansion remains limited by funding gaps (Jahnke et al., 2021). Similarly, Peru has increasingly emphasized mental health and psychosocial support within maternal and child health strategies, emphasizing detection of depression, anxiety and domestic violence during the pregnancy and postpartum period (Ministerio de Salud Del Peru (MINSA) and Dirección de Salud Mental, 2011).

Despite these efforts, both countries continue to face issues such as uneven resource distribution, and a rural–urban divide in service availability. Lima, the capital city of Peru, concentrates 70% of Peruvian psychiatrists (Rondon, 2009), which has led to continued reliance on a diverse mix of service providers, including actors from the private sector and non-governmental organizations. These challenges may be further compounded for marginalized populations, including refugees and minority groups. Ecuador hosts one of the largest displaced populations in Latin America, including long-term Colombian refugees and, more recently, Venezuelan migrants (UNHCR, the UN Refugee Agency, 2025), while Peru is home to over one million Venezuelans (GTRM-Perú, 2022). In both countries, migrants face economic insecurity, high out-of-pocket healthcare costs, and discrimination in clinical settings, limiting access to care (Pinto-Alvarez et al., 2024). Indigenous communities also experience longstanding structural inequities, including lower access to maternal health services, especially in rural areas (López-Cevallos and Chi, 2010). These disparities are compounded by cultural and linguistic barriers and a lack of integration between formal health systems and Indigenous healing practices (Bautista-Valarezo et al., 2021).

Given the diversity of actors and service providers whose work addresses or touches upon maternal mental health in Ecuador and Peru, the objectives of this study were to (1) identify barriers to care for perinatal mental health and (2) identify responses to these barriers, from the perspective of services providers from a range of organizations and institutions engaged in maternal mental health research and practice in these countries.

## Methods

### Study design

We conducted a two-phase study to understand the landscape of perinatal mental health in Ecuador and Peru. This approach combined organizational mapping (phase 1) and qualitative interviews (phase 2). The COREQ checklist was used to guide reporting (Tong et al., 2007).

*In the first phase*, we completed a mapping exercise to identify institutions and actors engaged in (1) maternal health (with or without mental health), (2) mental health (with or without a focus on women) and (3) maternal mental health specifically. We conducted internet searches using combinations of terms such as “maternal health,” “mental health,” “NGO” and country-specific terms including “Ecuador” and “Peru,” in both English and Spanish. Searches were conducted via Google, Google Scholar, PubMed and SciELO. A complete list of search terms is provided in Supplementary Table S3. Due to the large number of search results, we limited our review to the first 10 pages of search results (approximately 100 links per query), prioritizing those from known NGOs, academic institutions, government agencies and international organizations. Each entry was screened for relevance to the study’s objectives and assessed for credibility (relevance to either maternal health, mental health or both), geographic scope (Ecuador or Peru), focus population (women, either in the general population or any subgroup) and availability of programmatic information (mention of ongoing or recent activities relevant to the project). This was complemented by a hand search of websites from major international NGOs and UN agencies known to operate in Ecuador and Peru. The search and review process were conducted in English and Spanish by co-authors (OC, EF and JCV) familiar with mental health systems in each country. Excluded websites and organizations were not systematically tracked.

*In the second phase*, we contacted representatives from the organizations identified in the first phase for key informant interviews. Organizations/actors were invited to participate in interviews via email using the contact information listed on their websites. Given that international organizations often support policymakers and programmatic operations on these topics, actors were invited to participate even when they were not personally based in Ecuador or Peru, as long as their organization’s work directly impacted on programs or policies in either of the two countries (e.g., actors engaged in transnational or binational actions). We also expanded the pool of key informants by asking initial participants to suggest other relevant individuals or organizations not initially identified in the study’s first phase, and with existing contacts. Three interviewees were previously known to members of the study team through existing professional networks, while other interviewees were not known to the study team prior to the interview. Prior to the interview, we shared a consent form identifying the study team and describing our reasons for conducting the research.

Semistructured, in-depth interviews were conducted via Zoom in English or Spanish (depending on the preference of the interviewee) by faculty co-authors IF (PhD), and GOL (PhD), both of whom have prior training and experience in qualitative research and previously experience researching mental health and/or maternal health in the target countries, and student researchers EF (MPH) and NHL (MD), who had completed prior coursework in qualitative research but did not have prior interview experience. All four interviewers are women who were based in the United States at the time of data collection. IF and NHL are native Spanish speaker with fluency in English, and GOL and EF are native English speakers. Only interviewers and interviewees were present. We used an interview guide adapted from the WHO Mental Health and Psychosocial Support Assessment toolkit (World Health Organization, 2012). The interview guide aimed to explore providers’ roles, organizational capacities, linkages with other institutions and perceived barriers to care. The interview guide was not formally pilot tested. All interviews were recorded with the permission of the interviewee, or if the interviewee preferred not to be recorded (in 4 instances), detailed notes were taken by the interviewer. Members of the study team met to iteratively review transcripts and notes until saturation was research. Transcripts and interview notes were then anonymized. Interviews were transcribed, and Spanish interviews translated into English using translation software (Google Translate), which was then reviewed and corrected by the interviewer, using the interview audiorecording as a reference. Studies have demonstrated that this approach (machine translation-generated drafts, followed by human review), can expedite the translation process while maintaining accuracy (Vieira, 2019). Transcripts were not returned to interviewees for review. Analysis was conducted in English. Interviews lasted 30–60 min, and no repeat interviews were conducted.

## Analysis

After an initial read-through of the complete transcripts, interviews were analyzed using a framework-informed inductive approach (Fereday and Muir-Cochrane, 2006) by a single coder (OC) using NVivo software (version 14.23.2). Although only one researcher independently coded the data, the codebook, initial categories and emerging themes were shared with members of the study team, who provided feedback and endorsed the final structure. This served as a form of peer debriefing and contributed to methodological triangulation. Open-coding procedures were used to identify key themes and concepts related to structural barriers to care as well as solutions (implemented or proposed) to address these barriers. Subsequently, codes were redefined and grouped into thematic categories through axial coding, allowing for the emergence of overarching themes that encapsulate the essence of the main topics under investigation (as described in the interview guide in Supplementary Table S1). Following recommended practices for thematic analysis, analytic memos were employed throughout the process to facilitate deeper interpretation of the data and guide the iterative coding process about the research questions and conceptual framework (Klassen et al., 2016; Smith et al., 2019). A conceptual framework was used to further organize barriers to care across individual, family, and community levels, health system levels, and structural levels. This framework was based on that of Sambrook (Smith et al., 2019) and had been previously adapted to an LMIC context by (Brown and Sprague, 2021). We chose this framework because its’ focus on interaction between barriers at different levels of the health system (i.e., barriers experienced by individual patients, barriers experienced by providers engaged in care delivery, and political and economic barriers experienced by policymakers) reflected the diversity of interest holder viewpoints expressed through our interviews. To this, we added proposed solutions, which were organized according to their primary approach (broadly “top-down” and systemic or “bottom-up” and community-based).

Participants were not asked to review their transcripts to avoid placing an additional burden on them. Coding was completed in March 2025. The study codebook is provided in Supplementary Table S2 with a complete description of the definition for each code.

## Results

### Phase 1. Institutional mapping

We identified 30 interest holder organizations engaged in maternal health, mental health or some combination of the two in Ecuador and Peru. These interest holders included eight based in Peru, 21 based in Ecuador, and one US-based organization that operates in one or both countries. Among them, 13 focused primarily on maternal health, 12 on mental health, and 5 addressed both maternal and mental health.

Interest holders varied in scope and operational reach, with 3 working at the local or regional level, 18 at the national level, and 9 at the international level. Nongovernmental organizations (NGOs) and global development agencies comprised most of these interest holders, often focusing on vulnerable populations such as refugees, low- and middle-income families, women, children, Indigenous communities and migrants. These programs tended to focus on psychosocial support, mental health literacy, maternal care and community-based education.

### Phase 2. Qualitative interviews

A total of 47 individuals were initially contacted, including 19 based in Ecuador, 17 in Peru, 9 in the United States, 1 in Colombia and 1 in Canada. Of these, 17 participants were interviewed: 12 from Ecuador and 5 from Peru. Participant demographic information is outlined in Table 1. Most participants were female (n = 12). Participants represented providers from diverse sectors, including researchers (n = 8), government organizations (n = 2), NGOs (n = 5) and the private sector (n = 2).

**Table 1.** Demographic information of participants (n = 17)

Characteristics	n (%)
**Gender**	
Male	5 (29.41)
Female	12 (70.59)
**Location**	
Ecuador	12 (70.59)
Peru	5 (29.41)
**Role**	
Researcher	8 (47.06)
Government organization	2 (11.76)
NGO	5 (29.41)
Private sector	2 (11.76)

**Figure 1.**
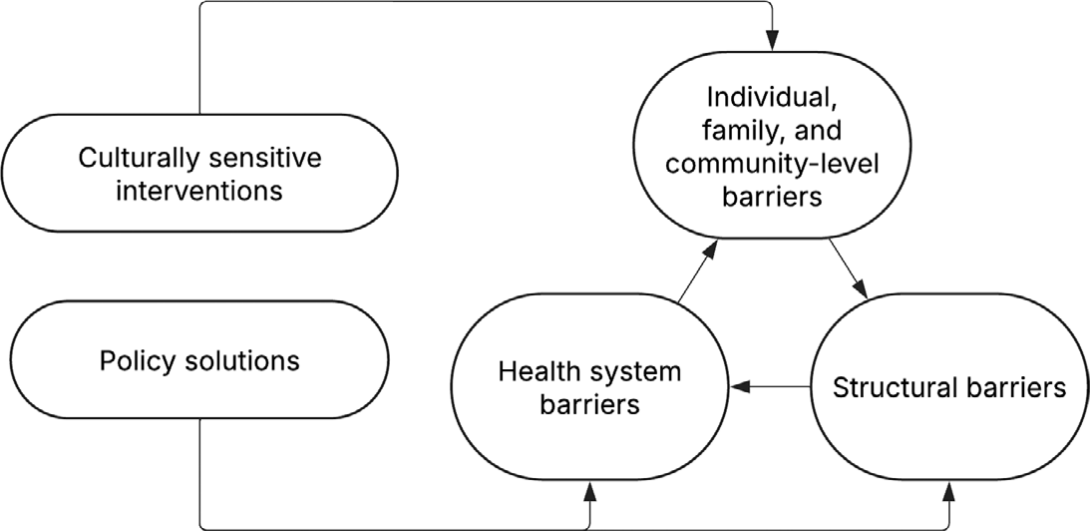
Conceptual framework of barriers and proposed solutions.

**Figure 2.**
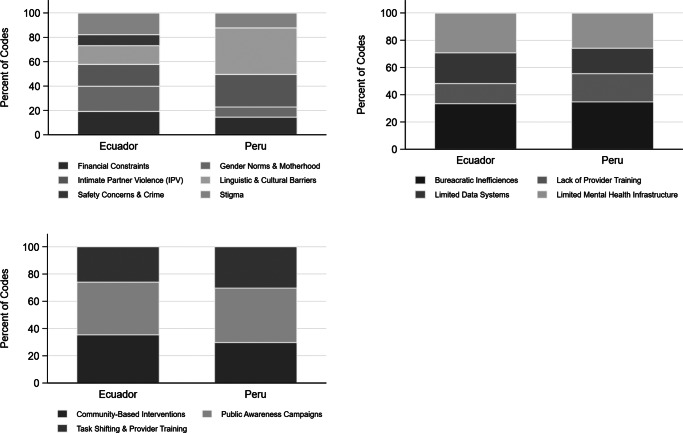
Relative frequency of barriers and proposed solutions between Peru and Ecuador. Culturally-sensitive solutions and policy solutions are combined in the third panel.

Results were organized by (1) challenges at the individual level, family and community (sociocultural) level, (2) health system and (3) structural challenges, as well as (4) proposed solutions (Smith et al., 2019; Brown and Sprague, 2021) (Figures 1 and 2).

#### Perceived challenges at the individual, family and community levels

Often coincided with issues such as stigma operating individually, within the family, and within the community, resulting in limited care-seeking behaviors and delayed access to maternal mental health care. Financial constraints and gender-based violence further limited access to care at the family level, while the unique challenges faced by certain groups, such as among Indigenous and migrant women, often limited access to care at the community level.

Participants shared that many women do not recognize or seek help for perinatal mental health issues like postpartum depression or anxiety, often because these aren’t seen as medical problems. Stigma around mental health and idealized views of motherhood, where there is little space to talk about sadness or stress, make it even harder for women to acknowledge or express what they are going through:

Postpartum depression is not given the importance that it has, even though it affects the mother and, logically, the mother-child relationship. But this is not openly expressed, including what the symptoms are and what a woman might feel. Why? Because in our Ecuadorian society, it’s like, ‘You can’t afford to be sad when you are taking care of a child and have so many responsibilities.’ Having a child is supposed to be a reason for happiness. So, a woman who feels sadness is going against those deeply ingrained social beliefs. (Participant #13, Ecuador)

These issues were exacerbated in the context of intimate partner violence, where women are prevented from seeking care. One provider described how this dynamic plays out in clinical settings:

Women many times said, ‘I didn’t know that I was suffering from some kind of violence. I couldn’t recognize it, for example, psychological violence. They couldn’t recognize it. And they started feeling bad. They felt anxious. They felt unworthy, but they didn’t recognize where it came from. (Participant #3, Ecuador)

What we’ve heard over and over again is that women, often if they are struggling or stressed, are prevented from actually accessing the services that they can use to support themselves. Controlling behaviors and gender-based violence can be a real barrier to women getting the care they need. (Participant #6, Ecuador)

For women and their families who acknowledged mental health symptoms and wanted to seek care, barriers related to financial costs, safety and language limited their ability to access care consistently. Even when mental health services were available in the community, with some facilities offering low-cost mental health services, providers reported that even modest fees remained prohibitive for many women:

I do believe that [mental health access] is intimately related to aspects of people’s economic income. Going one day for care means abandoning your job and not having someone to leave your other children with. (Participant #7, Ecuador)

Safety concerns were also an issue in communities where high crime rates, especially gang violence or street crime, deterred women seeking care because they feared traveling to clinics:

We were working in really insecure communities where women didn’t feel safe, even sometimes leaving their houses (Participant #6, Ecuador)

Providers also highlighted additional, unique challenges faced by rural, Indigenous, migrant and refugee women, with linguistic, cultural and mobility differences creating significant barriers to perinatal mental health care. Indigenous women were described as feeling uncomfortable in Western healthcare settings because the healthcare system was perceived as not aligned with their cultural beliefs or knowledge systems:

There is a significant percentage of the Indigenous population, and therefore, it is a population that has its own culture, customs, traditions, and language. The Western healthcare model is quite shocking. Many times, the health professional has a different conception from that of the pregnant woman, and there is no kind of communion between them. So, there can be a lot of shock, for example, when a woman does not feel comfortable with her language, because the professional speaks to her in Spanish, but her native language is Kichwa. (Participant #7, Ecuador)

Providers also talked about how migrant and refugee communities often deal with ongoing stress from difficult life circumstances and being excluded from systems of support. Many women in these communities face financial insecurity, displacement, discrimination and trouble accessing healthcare, all of which take a toll on their mental health:

Especially with the populations that I work with, there are often migrant or displaced populations. There’s a lot of structural adversity and chronic stress related to poverty, but also an inability to access certain systems and supports that can be really important to women, especially during this period of their life. (Participant #6, Ecuador)

There is a lot of stigma and xenophobia related to refugees coming into Ecuador.(Participant #3, Ecuador)

#### Health system barriers

Participants often pointed to staff shortages, disorganized services, and limited insurance coverage as significant barriers in the health system, issues tied to wider economic challenges. These problems were seen across both public and private healthcare. Public hospitals often do not have enough staff or proper facilities, while private care is too expensive for many people. Efforts to fix these issues are also held back by red tape and poor coordination between institutions.

One of the most consistent comments by participants was that maternal healthcare providers often lacked training in mental health. This, alongside a shortage of mental health specialists, led to inconsistent management of postpartum psychological distress and delays in recognizing symptoms and connecting patients to appropriate services.

They are too short [of] staff. There is very limited space to support women, and very few specialized people that can bring quality support and actually follow up in terms of mental health support. (Participant #3, Ecuador)

This concern was exacerbated for women in rural or underserved areas who often must travel long distances to reach healthcare facilities to be seen by a specialist:

The situation is widely different between cities, towns, and rural areas. You know, rural doctors, we are not prepared to do psychological support. We can recognize the problem, and we can give some advice, but not as psychologists. So, it is almost impossible to do. (Participant #5, Ecuador)

Beyond staff shortages, providers noted that there is a lack of tertiary referral hospitals or other specialized support for maternal mental health, which limits treatment options. One provider explained:

There is no national hospital for mental health care in Ecuador. In Peru, the main national reference center is Honorio Delgado in Lima, but it primarily handles the most complex psychiatric cases. Parents are referred there for treatment, but it belongs to the general health department rather than a specialized maternal mental health unit. (Participant #4, Peru)

Further challenges related to the limited coordination between maternal mental health and perinatal care stemmed from broader systemic gaps in health infrastructure, workforce and funding. Participants across settings described how these shortages affected both postpartum care and mental health service delivery:

Postpartum integration, for example, is really bad. No one knows what to do… So, it doesn’t matter if you have money or not, there are not enough resources to understand how to manage this better. (Participant #2, Ecuador)

Access to health services is limited. In the public or private, the same thing usually happens. Why? Because there is… a gap in human resources and in the capital itself. (Participant #4, Peru)

#### Structural barriers

The absence of clear governmental prioritization of mental health, gaps in regulatory oversight, bureaucratic inefficiencies and inconsistent or unreliable health data reporting were regarded by participants as contributing to a fragmented and ineffective approach to maternal mental health.

Providers widely agreed that maternal mental health is not a national health priority. Some described that, historically, external policy pressures, rather than internal priorities, have driven the maternal mental health agenda, leading to a lack of sustained accountability and minimal concrete improvements in mental health services:

Authorities in places like Ecuador and Peru don’t have enough information on how to prioritize maternal mental health. External pressures, like the Millennium Development Goals, have been instrumental in pushing authorities to make changes, but unless there is accountability, there’s no guarantee these goals will translate into real policy changes. (Participant #8, Ecuador)

As mental health professionals, we are not always taken seriously. For example, when we request resources, the response is often, ‘Okay, but what is that?’ or ‘We’re not comfortable talking about that.’ (Participant #2, Ecuador)

Providers also shared their frustration about how often mental health policies change, saying it shows a lack of steady commitment from the government to really address the issue:

We don’t have clear regulations in place. A few years ago, the Ministry’s Division for Mental Health was taken out entirely, and just last year, it was put back in place. But there are really, really few people working in it. The system lacks sufficient professionals, and to compensate, the government is outsourcing mental health services to private clinics. The problem is that many of these clinics don’t have qualified, specialized professionals; they are run more like businesses in some cases. (Participant #2, Ecuador)

One provider explained how efforts to establish maternal mental health programs require extensive political and administrative negotiations:

In the public sector, there is no rehabilitation infrastructure for maternal mental health. We are essentially entering new territory, which means that reaching these patients requires a lot of bureaucratic and political work, getting institutional review board (IRB) approvals, ensuring legal compliance, and securing permission to operate within hospitals. It’s an uphill battle before we can even begin offering care. (Participant #1, Ecuador)

An additional concern was limited access to mental health data, and the manipulation of maternal health data to give the false impression of progress, both of which may obscure the accurate scale of maternal mental health challenges and affect policy decisions and funding allocations. As one provider from Ecuador noted,

A major issue in practitioner leadership roles is the inconsistency of health data. In Ecuador, for example, we have seen manipulation of maternal mortality data, where denominators have been adjusted to make rates appear lower. This means that when authorities compare rates over decades, they may not be reflecting actual improvements in care but rather statistical adjustments that hide the reality. If you look closely at the calculations, the rates may not be changing due to better care but due to changes in how they are reported. (Participant #8, Ecuador)

## Solutions

Interest holders from different groups also shared ideas for solutions. Some have already been put into practice, while others are still more of a goal due to broader systems challenges.

### Culturally sensitive interventions

To reduce stigma and raise awareness, participants stressed the importance of offering both preventative and treatment-focused mental health services. They saw community-based care as necessary for recognizing and addressing mental health concerns early, before they become more serious:

And I think the main takeaway and why we started the work we did in Ecuador… really was the recommendation that we need to focus more on prevention and not only on response. (Participant #6, Ecuador).

They developed this really, really simple training program, where you train primary health workers, not specialists in mental health, so that way they could help people in the first stages of distress or do a really good referral. (Participant #2, Ecuador).

Community-led epidemiological surveillance was also highlighted as an essential strategy for reducing maternal health disparities. One participant emphasized the importance of involving local women in these efforts, with one participant describing an initiative in indigenous communities that aimed to bridge traditional and biomedical approaches to mental health:

We brought together traditional healers, doctors, and psychologists, not to impose one way over the other, but to have an open dialogue about mental health. It was about mutual learning, where both sides could share their perspectives on issues like suicide, domestic violence, and maternal well-being. (Participant #2, Ecuador).

Another successful strategy came from the use of community-based facilitators trained to deliver psychosocial support.

We couldn’t really find an intervention that fit the priorities of the women in Ecuador that we had interviewed. Those priorities were social support, kind of safety and community security, mental health, and kind of well-being more broadly, and community connectedness. (Participant #6, Ecuador).

This experience led to the co-creation of a community-led, group-based psychosocial program. The sessions, led by trained community members, directly addressed the social support, safety, and well-being concerns women had identified.

A third, hospital-based program partnered with a community-driven maternal health initiative to involve former patients in hospital decision-making, as intermediaries between hospitals and the community:

When the committees of moms are escalated to management, participating in the decision-making of the hospital, the hospitals do so much better (Participant #8, Ecuador).

It is greatly successful because their stake is not only them but their child, so their engagement is not artificial. It’s actually very meaningful (Participant #8, Ecuador).

Other recommendations involved expanding the role of primary health workers in providing maternal mental health support and integrating mental health support into other women’s health services, such as services related to preventing and addressing intimate partner violence interventions or services to provide stable housing to migrants.

### Health system and policy solutions

To reduce stigma, there was a perceived need for clear, evidence-based information to be shared through trusted channels, such as through official governmental television channels:

We need education for the whole population… through mass media to advertise that this can happen, and it’s not rare. The information must be about the symptoms and signs that women present with depression. Everyone, men and women, should understand that this is a real condition, not just laziness. (Participant #5, Ecuador)

In addition, incorporating greater maternal mental health education into routine healthcare visits for women was suggested to improve outcomes.

To address limited training and specialization to address mental health issues within the health care system, providing continuing education to health workers was regarded as a priority:

“The government should train nurses or assistant nurses who are from the local area, from the community. We have community health workers from the same community, so they can help narrow the doctor shortage. (Participant #5, Ecuador).

To increase governmental awareness and support for maternal mental health within the national and international policy agendas, they urged the international community to “be more inclusive” and aware that “the majority of the population… is not in high-income countries, but in middle- and low-income countries. (Participant #1, Ecuador).

At the national level, respondents also described the importance of bridging the gap between research and policy action through better data, including economic evaluations.

For inside Ecuador, maybe the suggestion would be doing more research and listening also to what the results are, and try to implement it. (Participant #1, Ecuador)

Let’s put a number on the losses. Let’s put a number on intimate partner violence. Let’s put a number on maternal mental health treatment. How much are we losing in care? How much are we losing in costs? (Participant #8, Ecuador).

## Discussion

This analysis looked at organizations in Ecuador and Peru that work in maternal and mental health to understand how they see the challenges and responses around perinatal mental health care. While several studies, including our own, have described experiences of women in both country (Miño and Flores, 2023; Forer et al., 2024; Zaman et al., 2024; Aranda et al., 2025) and some have described experiences of front-line healthcare providers (Cavero et al., 2018; Vargas et al., 2021), relatively fewer have engaged with institutional representatives who are indirectly engaged in the provision of care through the provision of programs and services. To complement prior work, we therefore aimed to understand challenges from this perspective.

Prior studies that describe the perspectives of women have often emphasized the importance of stigma, as well as economic and geographic barriers to care, often related to rurality or membership in a minority group. Studies focused on provider experiences have further emphasized the lack of trained providers, and lack of continuity or care, and challenges to collaboration between healthcare providers (Cavero et al., 2018). Our results at the patient and healthcare level echoed these findings, while additionally suggesting that, in both countries, services are often scattered and uncoordinated, offered by a mix of public agencies, private clinics, NGOs and community groups, that often fail to coordinate fully, in part due to limited funding and limited coordination by larger institutions.

Our findings reinforce the need to expand access to multilevel interventions to improve access to perinatal mental health care in Ecuador and Peru. This includes public awareness campaigns and mental health education programs to reduce stigma, improve symptom recognition, encourage early care-seeking behaviors (Lasater et al., 2017; Brown et al., 2020; World Health Organization, 2022), community education efforts to normalizing discussions about maternal mental health and increasing engagement with available services (Lasater et al., 2017; Brown et al., 2020; World Health Organization, 2022) and the integration of community-based and culturally appropriate care models to improve accessibility for marginalized groups, particularly Indigenous and migrant women (Lara-Cinisomo et al., 2014; Gallegos et al., 2017; Rondon, 2020).

At the organizational level, public health services in both countries are often understaffed, with limited mental health training for primary care physicians and a lack of mental health specialists and resources (Lasater et al., 2017; Brown et al., 2020; World Health Organization, 2022; Wilson et al., 2024). Addressing these workforce shortages requires further integrating mental health screening and support into standard maternal healthcare services (Lasater et al., 2017; Brown et al., 2020; World Health Organization, 2022; Wilson et al., 2024), healthcare providers to build mental health literacy, and utilizing task-shifting strategies to involve general health workers in perinatal mental health support (Lasater et al., 2017; Brown et al., 2020; Ng’oma et al., 2020). These approaches have been shown to be effective in expanding service delivery capacity in resource-limited settings, improving early identification of maternal mental health concerns and strengthening continuity of care (Lasater et al., 2017; Brown et al., 2020; World Health Organization, 2022; Wilson et al., 2024).

At the structural level, policy reforms ensuring adequate funding, service expansion and the formal inclusion of perinatal mental health within national healthcare priorities are critical steps toward closing existing gaps (Lara-Cinisomo et al., 2014; Rondon, 2020). Sustained investment in mental health infrastructure and workforce development is necessary to support the long-term sustainability of perinatal mental health services (Lara-Cinisomo et al., 2014; Rondon, 2020). Strengthening intersectoral collaboration among government agencies, NGOs, academic institutions and professional associations could also enhance service coordination and resource distribution, ultimately improving accessibility and care quality (Lara-Cinisomo et al., 2014; Rondon, 2020).

Most of the organizations identified through our search were based in cities and had more resources, likely because they have a more substantial presence online. This points to a bigger issue: maternal mental health services tend to be centered in urban areas, while rural and Indigenous communities often struggle to access care. As a result, the groups included in this analysis better reflect national and city-based efforts rather than local or rural initiatives.

This study has several limitations. The sample size was small, with 17 participants, including only 5 from Peru, limiting the diversity of perspectives captured, especially from areas with varied resources and sociocultural contexts. While the smaller number of interviewees from Peru limited our ability to conduct a comparative analysis of experiences between the 2 countries, interviewees discussed very similar themes, and we therefore chose to include these results in a pooled analysis. Nevertheless, our results may be less representative of the Peruvian context, which is unfortunate given other literature suggests that Peru’s recent progress toward increasing mental health coverage may be in part of greater coordination between governmental and nongovernmental actors (Eappen et al., 2018), as well as greater economic growth over the past decade. An additional challenge is that most participants were women, likely reflecting the demographic make-up of the maternal health field, but also potentially influencing results through an empathetic perspective or one focused on female psychosocial vulnerabilities. Finally, the emphasis on better-resourced, urban provinces may underrepresent challenges in underserved areas. Future research should therefore prioritize broader representation, especially from smaller, community-based organizations. Including more diverse interest holders may offer a fuller understanding of perinatal mental health care in Ecuador and Peru.

## Conclusion

Building on prior research, this study offers context-specific insights to inform perinatal mental health program design in Ecuador and Peru. We identified multilevel barriers to perinatal mental health care, including stigma, systemic fragmentation and limited health system capacity, and persistent inequities for rural, Indigenous and migrant populations, despite policy advances. Responses to these barriers included strengthening cross-sector collaboration, expanding culturally grounded community approaches, and integrating mental health into maternal and primary care services to support inclusive, sustainable strategies for improving maternal mental health across the region.

## Supporting information

10.1017/gmh.2025.10090.sm001 Culbert et al. supplementary material Culbert et al. supplementary material

## Data Availability

The interview data analyzed during this study are available from the corresponding author upon reasonable request, subject to institutional review board approval.

## Supplementary material

The supplementary material for this article can be found at http://doi.org/10.1017/gmh.2025.10090.
