# Supplementary material for: A landscape analysis of perinatal mental health delivery among stakeholders in Ecuador and Peru
Source: Glob Ment Health (Camb). 2026 Feb 19;13:e42. doi: 10.1017/gmh.2025.10090 (PMC13458860; doi:10.1017/gmh.2025.10090)
Supplement: Supplementary file 1 [file S2054425125100903sup001.zip › CulbertSupplementalTable3.docx]

Supplemental Table 3. Search Terms

| **Source** | **Search Term** |
| --- | --- |
| Google (English results from past 5 years) | (“Maternal health” OR “mental health”) AND (“Ecuador” OR “Peru” OR “South American” OR “Latin America”) AND (“Government” OR “Non-governmental” OR “Academic” OR “Research” OR “Program”); Filters applied: in the last 5 years, English |
| Google (Spanish results from the past 5 years) | (“Salud materna” OR “salud mental”) AND (“Ecuador” OR “Perú” O R“Sudamérica” OR “Latinoamérica”) AND (“Gobierno” OR “No gubernamental” OR “Académico” OR “Investigación” OR “Programa”); Filters applied: in the last 5 years, Spanish. |
| Pubmed | ("maternal mental health" OR "women's mental health" OR "women's health" OR "mental health") AND (Peru OR Ecuador); Filters applied: in the last 5 years, English. |
| Scielo.org | (“Salud materna” OR “salud mental”) AND (“Ecuador” OR “Perú” OR“Sudamérica” OR “Latinoamérica”) AND (“Gobierno” OR “No gubernamental” OR “Académico” OR “Investigación” OR “Programa”); Filters applied: in the last 5 years, Spanish |
