# Supplementary material for: A landscape analysis of perinatal mental health delivery among stakeholders in Ecuador and Peru
Source: Glob Ment Health (Camb). 2026 Feb 19;13:e42. doi: 10.1017/gmh.2025.10090 (PMC13458860; doi:10.1017/gmh.2025.10090)
Supplement: Supplementary file 1 [file S2054425125100903sup001.zip › CulbertSupplementalTable1.docx]

Supplemental Table 1. Interview Guide

| **Domains** | **Interview Questions** |
| --- | --- |
| Introduction | Thank you so much for taking the time to talk with me today! Before we get started, I want to just introduce myself a bit more. [interviewer gives brief introduction]. Do you mind giving me a brief introduction to yourself? Maybe include what got you interested in [relevant field]? |
| Purpose of Interview | Today we’re going to be talking about maternal health, especially mental health, during pregnancy and postpartum. The aim of these interviews is to start developing an understanding about factors impacting maternal health and maternal depression programing in Ecuador/Peru. I’m going to ask about your experiences in [X] field, and, as I mentioned earlier, you do not have to answer any questions you don’t want to. I will be recording this interview as well, so I want to confirm you are comfortable with that. Do you have any questions before I start recording?  [start recording] Okay, we are going to get started. Thank you for confirming I can record this. |
| Mental Health Professionals | - What are some of the mental health problems of women in the communities or populations you work in?   - Have these problems changed during your time in the field? - Can you tell me about programs and/or interventions for mental health you work on? - Do you link with other community-based organizations?   - If yes, how? What organizations do you link to? - What barriers do people face to participate in these programs/interventions?   - *Follow up:* Have you thought about any ways to minimize these challenges? - What are other things that work in communities to support women’s mental health? - Is there anything you wish was farther along with the [inset community of interest] when it comes to mental health? - Is there anything else that you would like to add?   - *If appropriate:* Do you know of anyone else who might be interested in speaking with us about some of these issues? |
| Maternal Health Professionals | - What are some of the challenges women face during and after pregnancy in the communities where you work?   - Have any of these problems changed during your time in the field? - Can you tell me about programs and/or interventions for pregnancy that you have worked on?   - What barriers do people face to participate in these programs/interventions?     - Have you thought about any ways to minimize these challenges? - What are other things that work in communities to support pre-conception, pregnancy, and postpartum? - Do you link with other community-based organizations?   - If yes, how? What organizations do you link to? - Is there anything you wish was further along with the [insert community of interest] when it comes to pregnancy? - Is there anything else that you would like to add?   - *If appropriate:* Do you know of anyone else who might be interested in speaking with us about some of these issues? |
| Social Climate | - What types of social problems do the communities/populations you work in face?   - *Probe:* what are other problems? Can you think of more?   - Which are the most important? - How do communities deal with the problems you mentioned to me? - What has your experience been implementing programs with the local governments? - How do you think people in the communities you work in view the current government? - What are some of the challenges faced when implementing programs in these communities? Can you think of ways to overcome them? - Can you tell me about the communities your organization works in? What characterizes them? - Do you link with other community-based organizations?   - If yes, how? What organizations do you link to? - Is there anything else that you would like to add?   - *If appropriate:* Do you know of anyone else who might be interested in speaking with us about some of these issues? |
| Postpartum Depression | - What types of problems do women in the pre- or postpartum period have?   - You mentioned a number of problems, including [READ OUT PROBLEMS NAMED ABOVE]. Of these problems, which do you think is the most important problem? “From your perspective, what makes this problem more important than other problems?”     - *Probe:* are there any other problems you can think of? - Have you noticed any changes in the problems you mentioned to me in the past five years? 10 years?   - If so, what have those looked like?   - If not, would you have expected any type of change/progression? Why or why not? - How do communities deal with the problems you mentioned to me? - Can you tell me about the interventions/programs you have worked with that target pre- or postpartum depression? - What are the challenges faced when implementing these interventions/programs? - What types of barriers do people face to participate in these programs? - What would be needed to make interventions/programs for pre- or postpartum depression more successful? - Is there anyone else you know of that might be willing to connect with us over some of these questions/issues? |
