# Supplementary material for: A landscape analysis of perinatal mental health delivery among stakeholders in Ecuador and Peru
Source: Glob Ment Health (Camb). 2026 Feb 19;13:e42. doi: 10.1017/gmh.2025.10090 (PMC13458860; doi:10.1017/gmh.2025.10090)
Supplement: Supplementary file 1 [file S2054425125100903sup001.zip › CulbertSupplementalTable2.docx]

Supplemental Table 2. Codebook

1. Challenges at the Individual, Family, and Community Level

| **Code** | **Definition** | **Inclusion Criteria** | **Exclusion Criteria** | **Example Quote** |
| --- | --- | --- | --- | --- |
| Stigma | Negative perceptions or societal attitudes preventing women from seeking mental health care. | Mentions of self-stigma, cultural stigma, or fear of judgment. | General discussions about mental health without stigma-related barriers. | "There is still a lot of stigma regarding mental health. Women many times said, ‘I didn't know that I was suffering from some kind of violence. I couldn't recognize it, for example, psychological violence. They couldn't recognize it. And they started feeling bad. They felt anxious. They felt unworthy... but they didn't recognize where it came from.’" |
| Gender Norms & Motherhood | Cultural expectations that pressure women to idealize motherhood, leaving little room for mental health struggles. | Discussion of societal norms about women’s roles affecting care-seeking. | Non-gendered discussions about barriers to care. | “In our Ecuadorian society, it’s like, ‘You can’t afford to be sad when you are taking care of a child and have so many responsibilities.’ Having a child is supposed to be a reason for happiness. So, a woman who feels sadness is going against those deeply ingrained social beliefs." |
| Financial Constraints | Economic difficulties preventing women from accessing mental health care. | Mentions of cost-related barriers, inability to leave work, lack of childcare. | Financial barriers unrelated to healthcare (e.g., housing, food). | "I do believe that [mental health access] is intimately related to aspects of people's economic income. Going one day for care means abandoning your job and not having someone to leave your other children with." |
| Intimate Partner Violence (IPV) | Abuse and controlling behaviors that prevent women from seeking mental health care. | Mentions of physical, emotional, or psychological violence affecting mental health. | General discussions of violence not related to health care access. | "What we’ve heard over and over again is that women, often if they are struggling or stressed, are prevented from actually accessing the services that they can use to support themselves. Controlling behaviors and gender-based violence can be a real barrier to women getting the care they need." |
| Safety Concerns & Crime | Fear of traveling to health facilities due to crime or violence. | Mentions of gang violence, street crime, or unsafe travel. | Safety concerns not related to healthcare access. | "We were working in really insecure communities where women didn't feel safe even sometimes leaving their houses." |
| Linguistic & Cultural Barriers | Language and cultural mismatches between providers and patients, especially for Indigenous and migrant women. | Discussions about discomfort in Western healthcare settings, lack of interpretation services. | Barriers unrelated to language or cultural factors. | "There is a significant percentage of Indigenous population, and therefore it is a population that has its own culture, customs, traditions, and language. The Western healthcare model is quite shocking. Many times, the health professional has a different conception than that of pregnant women, and there is no kind of communion between them. So, there can be a lot of shock, for example, when a woman does not feel comfortable with her language, because the professional speaks to her in Spanish, but her native language is Kichwa." |

2. Health System & Structural Barriers

| Code | Definition | Inclusion Criteria | Exclusion Criteria | Example Quote |
| --- | --- | --- | --- | --- |
| Lack of Provider Training | Deficiencies in maternal mental health training among healthcare providers. | Mentions of gaps in provider education, lack of mental health specialists. | General discussions about mental health care. | "They are too short [of] staff. There is... very limited space to support women, and very few specialized people that can bring quality support and actually follow up in terms of mental health support." |
| Limited Mental Health Infrastructure | Inadequate mental health facilities, staff shortages, and poor integration into maternal care. | Mentions of hospital limitations, referral system gaps. | Discussions unrelated to system capacity or workforce issues. | "There is no national hospital for mental health care in Ecuador. In Peru, the main national reference center is Honorio Delgado in Lima, but it primarily handles the most complex psychiatric cases. Parents are referred there for treatment, but it belongs to the general health department rather than a specialized maternal mental health unit." |
| Bureaucratic Inefficiencies | Administrative obstacles that delay or prevent maternal mental health service improvements. | Discussions of slow or ineffective policy implementation. | General government challenges not related to healthcare. | "Authorities in places like Ecuador and Peru don’t have enough information on how to prioritize maternal mental health. External pressures, like the Millennium Development Goals, have been instrumental in pushing authorities to make changes, but unless there is accountability, there’s no guarantee these goals will translate into real policy changes." |
| Poor Health Data Systems | Lack of reliable mental health data affecting policy decisions. | Mentions of inaccurate, manipulated, or missing health data. | Data issues unrelated to maternal mental health | "A major issue in practitioner leadership roles is the inconsistency of health data. In Ecuador, for example, we have seen manipulation of maternal mortality data, where denominators have been adjusted to make rates appear lower. This means that when authorities compare rates over decades, they may not be reflecting actual improvements in care but rather statistical adjustments that hide the reality. If you look closely at the calculations, the rates may not be changing due to better care but due to changes in how they are reported." |

3. Proposed Solutions

| Code | Definition | Inclusion Criteria | Exclusion Criteria | Example Quote |
| --- | --- | --- | --- | --- |
| Community-Based Interventions | Locally driven programs to promote awareness, provide social support, and bridge traditional and biomedical healthcare. | Mentions of Indigenous knowledge, community health workers, peer support programs. | Top-down policy changes. | “Maternal health interventions work best when they incorporate the empowerment of local women. But nothing works without resources, these programs must go hand-in-hand with state support.” |
| Task-Shifting & Provider Training | Training non-specialists, such as nurses or community health workers, to provide mental health support. | Discussions about upskilling providers and integrating mental health into routine care. | Solutions that only involve specialized professionals. | “The government should train nurses or assistant nurses who are from the local area, from the community. We have community health workers from the same community, so they can help narrow the doctor shortage.” |
| Public Awareness Campaigns | Mass media and public education efforts to reduce stigma and encourage care-seeking. | Mentions of using television, social media, or community education to promote awareness. | Discussions about stigma without specific public outreach solutions. | “We need education for the whole population… through mass media to advertise that this can happen, and it’s not rare. The information must be about the symptoms and signs that women present with depression. Everyone, men and women, should understand that this is a real condition, not just laziness.” |
