# Supplementary material for: A landscape analysis of perinatal mental health delivery among stakeholders in Ecuador and Peru
Source: Glob Ment Health (Camb). 2026 Feb 19;13:e42. doi: 10.1017/gmh.2025.10090 (PMC13458860; doi:10.1017/gmh.2025.10090)
Supplement: Peer review history

# Reviewer report: Review: A landscape analysis of perinatal mental health delivery among stakeholders in Ecuador and Peru — R0/PR2

**DOI:** 10.1017/gmh.2025.10090.pr2

**Authors:** Alexandra Meneses

**Affiliations:** Psychology, Universidad de Las Americas Facultad de Ciencias de la Salud, Ecuador

1. The title must align with the focus on perinatal mental health in women.

2. The objective must correspond to the title. In the abstract, it is stated: “This study examines healthcare providers’ perceptions of barriers to perinatal mental health care and the solutions they propose based on their experience in maternal mental health research and service delivery.”

3. The paragraph structure should be improved to follow the journal’s format. Some paragraphs have more spacing than others. Additionally, each paragraph should be no longer than 20 lines; please review and split those that appear too lengthy and exhaustive.

4. Regarding the theoretical review, some references need to be updated. The Ecuadorian Ministry of Public Health has issued guidelines promoting humanized childbirth. The ESAMyN program has proven beneficial, yet there appears to be little emphasis on this maternal and child health service provider in the manuscript.

5. Concerning the methodology, some gaps remain. It is unclear whether the mapped actors included only institutional officials or if women who use these services were also considered.

6. In the first phase of the methodology section, consulted databases are mentioned; however, the inclusion and exclusion criteria for the systematic review are not specified, nor is the screening process or search equation detailed.

7. It is not entirely clear whether informants who are not based in Ecuador or Peru have provided relevant data on the topic. The rationale behind their inclusion is unclear, and selecting representatives from the actual countries might have been more appropriate.

8. The contributions from healthcare providers should be better organized. There is a notable predominance of direct quotations related to the Ecuadorian context, with less focus on the reality in Peru.

9. The discussion section could be expanded based on the theoretical background.

10. Overall, the study requires better organization of ideas. It addresses two countries simultaneously on a highly complex issue, and significant gaps remain regarding maternal and child health in the Ecuadorian context. Additionally, based on the information provided, it seems that the informants were professionals working in the field, yet the perspectives of the women actually experiencing these issues were not considered—this could have significantly enhanced the impact of the study.

# Reviewer report: Review: A landscape analysis of perinatal mental health delivery among stakeholders in Ecuador and Peru — R0/PR3

**DOI:** 10.1017/gmh.2025.10090.pr3

**Authors:** Mateo Alejandro Andrade Miño

**Affiliations:** UTE University, Ecuador

1. This study has many strengths; it clearly presents the issue of perinatal maternal mental health in Ecuador and Peru, successfully identifying relevant barriers classified at different levels: individual, community, health system, and structural. Through interviews, proposals to address these barriers were collected, and recommendations aimed at improving the accessibility, quality, and sustainability of services are presented.

• It is recommended to state the study objectives more explicitly within the text to facilitate their identification by the reader.

2. Regarding the methodological approach, the article uses an inductive thematic analysis but does not mention the use of any checklist or standardized criteria (such as COREQ, SRQR, or CASP) to ensure the quality of the qualitative study. Including or declaring these frameworks would strengthen methodological transparency, facilitate critical appraisal, and increase the study’s credibility.

• It is recommended to include in the manuscript whether such a tool was used; if not, to explain the reason.

3. Concerning the sample, 17 interviewees were included: 12 from Ecuador and only 5 from Peru. Given that the health systems in both countries have significant differences, it is suggested to consider incorporating a comparative analysis between national contexts or discuss in the limitations how this imbalance could affect the validity and transferability of the recommendations across countries.

4. The sample shows a predominance of female participants. While this may reflect the reality of the maternal health field, it would be pertinent to reflect on how this composition might have influenced the results, potentially providing a more empathetic perspective or one focused on female psychosocial vulnerabilities.

5. Regarding visual elements, Figure 1 (“Conceptual framework of barriers and proposed solutions”) is mentioned but neither developed nor explained in depth within the main text.

• It is recommended to contextualize it and describe its analytical utility.

6. The study includes interviews conducted in Spanish that were translated into English for analysis. However, the translation process is not described: who performed it, under what criteria, and whether any cross-verification took place. Since qualitative analysis heavily depends on language and its nuances, this omission represents a possible risk of interpretative bias.

• It is recommended to detail the translation procedure, specifying if professional translators, native speakers, or any quality control measures were involved.

The study addresses a relevant issue with an appropriate approach and a well-structured thematic analysis. It contributes applicable knowledge for the design of public policies as well as the implementation of perinatal mental health programs in Latin America. Areas for improvement are identified in terms of methodological transparency, critical reflection on the sample, and justification of technical procedures such as translation. With adjustments in these aspects, the manuscript would be significantly strengthened for publication.

# Editor report: Recommendation: A landscape analysis of perinatal mental health delivery among stakeholders in Ecuador and Peru — R0/PR4

**DOI:** 10.1017/gmh.2025.10090.pr4

**Affiliations:** School of Public Health and Preventive Medicine, Monash University Faculty of Medicine Nursing and Health Sciences, Australia

Please address all the revisions suggested by the reviewers.

# Editor report: Decision: A landscape analysis of perinatal mental health delivery among stakeholders in Ecuador and Peru — R0/PR5

**DOI:** 10.1017/gmh.2025.10090.pr5

**Affiliations:** Johns Hopkins University Bloomberg School of Public Health, United States

No accompanying comment.

# Reviewer report: Review: A landscape analysis of perinatal mental health delivery among stakeholders in Ecuador and Peru — R1/PR7

**DOI:** 10.1017/gmh.2025.10090.pr7

**Authors:** Alexandra Meneses

**Affiliations:** Psychology, Universidad de Las Americas Facultad de Ciencias de la Salud, Ecuador

1. Title

The current title, “A Landscape Analysis of Perinatal Mental Health Delivery Among Stakeholders in Ecuador and Peru,” offers a general overview. However, I recommend refining it to better reflect the central objective of the study and ensure that the key research variables are clearly aligned with that objective. This adjustment will help readers more easily grasp the scope and analytical focus of the work. Consider incorporating elements such as barriers, key actors, and proposed solutions to strengthen the connection between the title and the study’s aims.

2. Method

It is important to clarify why the authors chose Google Translator to translate the interview transcripts. What evidence supports the validity of this tool for document translation in research contexts? Although the authors describe a mixed-methods approach, the structure suggests a combination of literature analysis and qualitative components. I recommend organizing the methodology section more clearly, specifying each design and the techniques applied.

3. Results

The results could be better synthesized, particularly the survey data collected. It would be helpful to present more visual summaries that distill key themes from the collective narratives—such as common concerns and converging solutions. This would enhance clarity and impact, especially for readers seeking actionable insights. The use of qualitative data analysis software such as MAXQDA or ATLAS.ti could have supported this effort, offering tools for thematic mapping and visual representation of patterns across participant responses.

4. Conclusions

I suggest structuring the conclusions to explicitly respond to each research objective, with one conclusion per section. This should consist of a concise summary and reflection by the authors. Citations are not necessary in this section and can be reserved for the discussion.

# Reviewer report: Review: A landscape analysis of perinatal mental health delivery among stakeholders in Ecuador and Peru — R1/PR8

**DOI:** 10.1017/gmh.2025.10090.pr8

**Authors:** Mateo Alejandro Andrade Miño

**Affiliations:** UTE University, Ecuador

Comments to the Author:

Point 1, regarding the statement of the study objectives within the text, has been adequately addressed.

Point 2, could be further strengthened by clearly indicating that the “COREQ checklist was used to guide the qualitative methodology” on the text

Points 3 / 4, have been properly resolved by including additional details in the Introduction and Limitations sections about sample composition and methodological considerations.

Point 5, the rationale for the chosen conceptual framework was explained

Point 6, specifying that the interviewer has English as a second language could provide additional clarity.

Overall, with these revisions and clarifications, we consider the manuscript suitable for publication.

# Editor report: Recommendation: A landscape analysis of perinatal mental health delivery among stakeholders in Ecuador and Peru — R1/PR9

**DOI:** 10.1017/gmh.2025.10090.pr9

**Affiliations:** School of Public Health and Preventive Medicine, Monash University Faculty of Medicine Nursing and Health Sciences, Australia

Please address all the revisions suggested by the reviewers. In addition, the term “Stake holder” is now contested and regarded as indicative of colonization. Please replace it with a more appropriate term. For example: interest holder.

# Editor report: Decision: A landscape analysis of perinatal mental health delivery among stakeholders in Ecuador and Peru — R1/PR10

**DOI:** 10.1017/gmh.2025.10090.pr10

**Affiliations:** Johns Hopkins University Bloomberg School of Public Health, United States

No accompanying comment.

# Reviewer report: Review: A landscape analysis of perinatal mental health delivery among stakeholders in Ecuador and Peru — R2/PR12

**DOI:** 10.1017/gmh.2025.10090.pr12

**Authors:** Alexandra Meneses

**Affiliations:** Psychology, Universidad de Las Americas Facultad de Ciencias de la Salud, Ecuador

Congratulations to the authors for the substantial improvements made to their study. I only suggest refining the manuscript in terms of style and formatting, aligning it with the journal’s author guidelines, once all reviewer comments have been addressed.

# Reviewer report: Review: A landscape analysis of perinatal mental health delivery among stakeholders in Ecuador and Peru — R2/PR13

**DOI:** 10.1017/gmh.2025.10090.pr13

**Authors:** Mateo Alejandro Andrade Miño

**Affiliations:** UTE University, Ecuador

The authors have carefully addressed all reviewer comments, resulting in clear improvements in the manuscript’s structure, clarity, and methodological transparency. The title has been refined, terminology updated, and the methodology section clarified.

The inclusion of co-author Natalia Halpern-Lagos is justified, its inclusion should be approved by the editor.

I consider the manuscript ready for publication in its current form. It represents a valuable contribution to the understanding of maternal mental health systems in Latin America.

# Editor report: Recommendation: A landscape analysis of perinatal mental health delivery among stakeholders in Ecuador and Peru — R2/PR14

**DOI:** 10.1017/gmh.2025.10090.pr14

**Affiliations:** School of Public Health and Preventive Medicine, Monash University Faculty of Medicine Nursing and Health Sciences, Australia

Thank you for submitting the revised paper, which I am pleased to accept.

# Editor report: Decision: A landscape analysis of perinatal mental health delivery among stakeholders in Ecuador and Peru — R2/PR15

**DOI:** 10.1017/gmh.2025.10090.pr15

**Affiliations:** Johns Hopkins University Bloomberg School of Public Health, United States

No accompanying comment.
